# Tobacco Use in Schizophrenia: A Literature Review

**DOI:** 10.1192/bjo.2025.10166

**Published:** 2025-06-20

**Authors:** Nishikant Thorat, Soham Kulkarni

**Affiliations:** 1 Byramjee Jeejeebhoy Government Medical College and Sassoon General Hospitals, Pune, India; 2 Independent Researcher, Pune, India

## Abstract

**Aims:** Schizophrenia is a mental illness with chronic course and varied outcomes, characterized by positive, negative, affective and cognitive symptoms along with aggression. Tobacco use is notably more prevalent in individuals with schizophrenia, often accompanied by severe dependence, compared with the general population. This literature review aims to explore the neurobiological mechanisms underlying tobacco use in schizophrenia, as well as potential treatment options and their associated benefits to the individual.

**Methods:** A comprehensive search was conducted on PubMed using the keywords “Tobacco use” and “Schizophrenia”. Information from Free Full-text articles, including systematic reviews, meta-analyses, clinical trials, randomized controlled trials, review articles and books and documents published within the last 10 years were included, and studies published in languages other than English were excluded.

**Results:** The prevalence of tobacco use in patients with schizophrenia is 45–88% compared with less than 16% of general population. Nicotine acts via Nicotinic Acetylcholine Receptors (nAChRs) modulating the release of neurotransmitters. It helps improve the connectivity between salience network and other brain regions such as ventrolateral prefrontal cortex and superior parietal lobule, amongst others, which are deficient in schizophrenia.

The self-medication hypothesis suggests that tobacco reduces cognitive deficits. It also reduces extrapyramidal symptoms by inducing cytochrome P450 1A2, interacting with nAChRs in the ventral tegmental area, and inhibiting monoamine oxidase enzymes, which helps counteract dopamine reduction caused by antipsychotics.

The addiction vulnerability hypothesis suggests that genetic, neurobiological, and environmental factors in schizophrenia also increase susceptibility to tobacco use. Animal model studies also suggest that developmental limbic abnormalities which are seen in schizophrenia could also alter behaviour associated with drug use.

From a prognostic point of view, tobacco use in schizophrenia significantly increases the risk of cardiovascular diseases, shortening lifespan by up to 25 years, and raises the likelihood of metabolic syndrome. Pharmacotherapies like varenicline, bupropion (sustained release), nicotine replacement therapies (NRT), and combinations of bupropion and NRT have shown some success. Electronic cigarettes, along with psychological approaches like Acceptance and Commitment Therapy, Mindfulness, and Contingency Management (both digital and in-person), show promise. Neuromodulation via transcranial magnetic stimulation has shown some promise with limited results.

**Conclusion:** It is seen that tobacco use in schizophrenia is influenced by genetic, neurobiological, and cognitive factors, with nicotine causing long-term health risks and decreased effectiveness of treatment, hence proper understanding is essential for adequate patient care.

